# Quantitative Structure–Activity Relationship Study of Bitter Di-, Tri- and Tetrapeptides Using Integrated Descriptors

**DOI:** 10.3390/molecules24152846

**Published:** 2019-08-05

**Authors:** Biyang Xu, Hau Yin Chung

**Affiliations:** Food and Nutritional Sciences Programme, School of Life Sciences, The Chinese University of Hong Kong, Shatin, New Territories, Hong Kong SAR, China

**Keywords:** QSAR, bitter, peptides, amino acid descriptors

## Abstract

New quantitative structure–activity relationship (QSAR) models for bitter peptides were built with integrated amino acid descriptors. Datasets contained 48 dipeptides, 52 tripeptides and 23 tetrapeptides with their reported bitter taste thresholds. Independent variables consisted of 14 amino acid descriptor sets. A bootstrapping soft shrinkage approach was utilized for variable selection. The importance of a variable was evaluated by both variable selecting frequency and standardized regression coefficient. Results indicated model qualities for di-, tri- and tetrapeptides with R^2^ and Q^2^ at 0.950 ± 0.002, 0.941 ± 0.001; 0.770 ± 0.006, 0.742 ± 0.004; and 0.972 ± 0.002, 0.956 ± 0.002, respectively. The hydrophobic C-terminal amino acid was the key determinant for bitterness in dipeptides, followed by the contribution of bulky hydrophobic N-terminal amino acids. For tripeptides, hydrophobicity of C-terminal amino acids and the electronic properties of the amino acids at the second position were important. For tetrapeptides, bulky hydrophobic amino acids at N-terminus, hydrophobicity and partial specific volume of amino acids at the second position, and the electronic properties of amino acids of the remaining two positions were critical. In summary, this study not only constructs reliable models for predicting the bitterness in different groups of peptides, but also facilitates better understanding of their structure-bitterness relationships and provides insights for their future studies.

## 1. Introduction

Food-derived peptides refer to various short amino acid sequences, normally comprised of 3–20 amino acids, originating from food protein [1,2]. They have attracted huge attention because of their great benefits to our cardiovascular, nervous, immune, and nutritional systems, e.g., anti-inflammation, anti-oxidant, and anti-hypertensive effects [3]. However, despite of their excellent health-promoting activities, their sensory attributes, especially the undesirable sensation like bitterness, should also be taken into consideration when using them as food additives. Bitterness is one of the five basic tastes and usually taken as an aversion to avoid toxic substances by mammals [4]. In fact, bitterness is frequently generated during enzymatic process to produce bioactive protein hydrolysates [5]. For example, most bioactive peptides, having inhibition effects of angiotensin-I-converting enzyme (ACE) to decrease blood pressure, elicit bitter taste [6,7]. Thus, understanding the bitterness in peptides is essential for effective use of peptides as food additives.

With high efficiency and low cost, bioinformatic approaches have become more and more popular in peptide research, especially the quantitative structure–activity relationship (QSAR) study, which is a basic tool to search for information relating chemical structure to biological activities [8]. For a QSAR model, a set of numerical descriptors related to the structure of interest, e.g., amino acids, serves as independent variables (X), while the biological activities are the dependent variables (Y). The relationship between the X and Y is built by using different methods like multiple linear regression (MLR), partial least square (PLS) regression, support vector machine (SVC), artificial neural network (ANN), etc. [9,10]. The earliest amino acid descriptor set reported in the literature is the “3z-scale”, which was obtained from 29 physiochemical properties of 20 coded-amino acids [11]. Three parameters, including z1, z2, and z3, were generated, representing the hydrophobicity, bulkiness/molecular size and electronic property of amino acids, respectively [11]. Since then, numerous amino acid descriptor sets generated from different properties of amino acids have been developed, e.g., the “ISA-ECI (Isotropic Surface Area and Electronic Charge Index) ” descriptor set connected with the isotropic surface area and the electronic index; the “DPPS (Divided Chemical Property Scores) ” descriptor set related to the electronic, steric, hydrophobic properties and hydrogen bond of amino acids, etc. [12,13]. Moreover, the properties of amino acids described by a single parameter have become more and more complicated, e.g., the second parameter of E descriptor set (E-2) is related to 10 properties of amino acids, including the conformational parameter for β-turn, the normalized frequency of turn, etc. [14]. Studies on the structure–bitterness relationship of peptides are limited when compared with works on other bioactivities like the ACE-inhibition effects [15]. In addition, most of the reported studies focused on the bitterness of dipeptides, with even less reports on tri- and tetrapeptides [5,6,7,15,16,17,18,19,20]. On the other hand, most reported QSAR models for bitterness relied on only one amino acid descriptor, which probably lack sufficient descriptive power and neglect the relationship between different descriptors.

As a result, this study aimed to (1) build reliable QSAR models to predict bitterness of di-, tri- and tetrapeptides by using an integration of 14 amino acid descriptors; and (2) find out the key factors that contribute to the bitterness of peptides. We did not study peptides comprised of more than four amino acids because of the limitation of available published data.

## 2. Results

### 2.1. QSAR Models for Bitter Taste di-, tri- and Tetrapeptides Using Integrated Descriptors

QSAR models were built to predict the bitter taste threshold of di-, tri- and tetrapeptides by using PLS regression. Datasets for di-, tri- and tetrapeptides were shown in Tables S1–S3. The descriptors included a combination of 14 amino acid descriptor sets. All of the details are shown in the Material and Methods (Section 4). 

The statistical parameters determined from the QSAR models for di-, tri- and tetrapeptides using integrated descriptor sets (a combination of 14 amino acid descriptor sets), with and without the bootstrapping soft shrinkage (BOSS) variable selection process are shown in Table 1. “A” is the number of principle components used in PLS regression. “R^2^” (coefficient of determination) and “RMSE” (root mean square error) indicate the fitting performance, with R^2^ the larger, the better, RMSE the smaller, the better. “Q^2^” (the cross-validated R^2^), and “RMSECV” (root mean square error cross-validation) demonstrate the predictive ability of a model, with Q^2^ the larger, the better, while RMSECV the smaller, the better.

For di-, tri- and tetrapeptides models without the BOSS variable selection process, their R^2^ and Q^2^ were at 0.948 and 0.874; 0.760 and 0.521; and 0.965 and 0.682, respectively. Although the model quality for dipeptides was good (with high R^2^ and Q^2^, low RMSECV and RMSE), model qualities for tri- and tetrapeptides were less ideal, particularly for the tripeptides with the values of Q^2^ close to 0.5, and RMSE near 0.3.

Thus, the BOSS variable selection method was used to further improve the models. In this study, the BOSS was run 100 times, and the results were expressed as mean ± SD (Table 1). After the variable selection, model qualities for all data sets were better, with R^2^ and Q^2^ for di-, tri- and tetrapeptides of 0.950 ± 0.002 and 0.941 ± 0.001; 0.700 ± 0.006 and 0.742 ± 0.004; and 0.972 ± 0.002 and 0.956 ± 0.002, respectively. The RMSECV also decreased dramatically for all three models and a moderate improvement of RMSE could be seen (Table 1). Moreover, compared with R^2^, the increase of Q^2^ was more evident for all of the models, suggesting the capability of the BOSS variable selection process to improve the predictability by the models.

**Figure 1:**
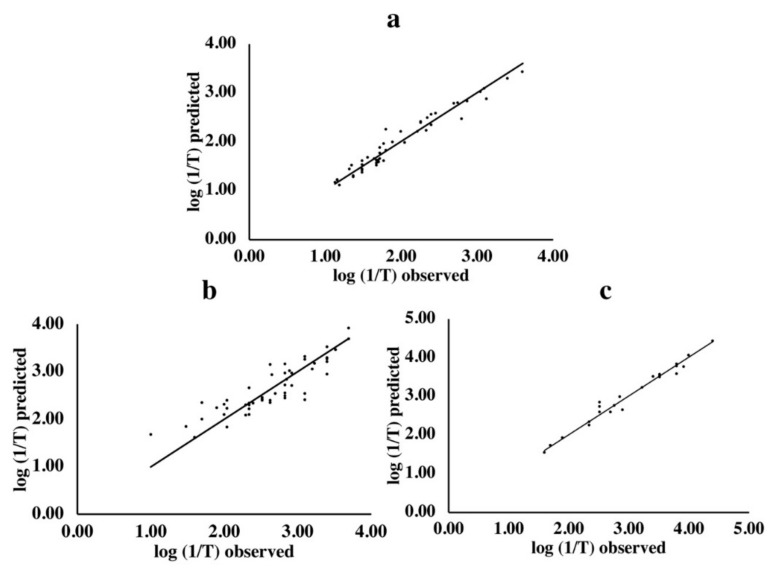
Observed vs. predicted bitter activities of di- (**a**), tri- (**b**) and tetrapeptides (**c**). The *x*-axis represents the observed sensory values from literature. The *y*-axis represents the corresponding predicted values derived from the model having the lowest root mean square error cross validation (RMSECV) obtained by 100 bootstrapping soft shrinkage (BOSS) runs.

The observed and predicted bitter activities were also compared (Figure 1). The predicted values were obtained from the models which had the smallest RMSECV and largest predictability (Q^2^) obtained by 100 BOSS runs. The observed values were based on previous human sensory evaluations [5]. Results indicated that the predicted bitter activities by this model were close to the observed ones for di- (Figure 1a), tri- (Figure 1b) and tetrapeptides (Figure 1c).

### 2.2. QSAR Models for Bitter Di-, Tri- and Tetrapeptides Using a Single Set of Amino Acid Descriptor

QSAR models built by integrated descriptor sets were compared with those models built by single descriptor sets (Table 2, Table 3 and Table 4). Although QSAR models for bitter di-, tri- and tetrapeptides were built with some of these descriptors before, methodologies and statistical parameters used were usually different. Besides, not all the descriptors included in this study have been utilized before for model development, especially for tri- and tetrapeptides. Thus, in order to have more reliable comparison results, 14 QSAR models based on single descriptor set were built again for each dataset in this study. Statistical results for di-, tri-, and tetrapeptides are shown in Table 2, Table 3 and Table 4 respectively. For the models obtained by 100 BOSS runs, both the average statistical parameters for the 100 BOSS runs (ID + BOSS1) and the statistical parameters for the ones with the smallest RMSECV from the 100 runs (ID + BOSS2) are shown (Table 2, Table 3 and Table 4).

With the Q^2^ and R^2^ values larger than 0.7, RMSECV and RMSE values lower than 0.3, most models for dipeptides built with single set of amino acid descriptor showed good predictive and fitting performances, and model with VHSE (Principle Components Score Vectors of Hydrophobic, Steric, and Electronic Properties) as descriptor set performed the best (Table 2). On the contrary, QSAR models for tripeptides using single descriptor set showed poor performance, e.g., most of the Q^2^ values are lower than 0.5, and RMSE values are larger than 0.3 (Table 3). Among them, models built by FASGI (Factor Analysis Scale of Generalized Amino Acid Information) and HESH (Hydrophobic, Electronic, Steric and Hydrogen) descriptor set were the better ones. Poor predictivity were also observed in models for tetrapeptides, but with the better ones went to that with HESH descriptor set (Table 4). In short, even without the variable selection process for the three datasets, models built with the integrated descriptor sets were comparable to those built with single descriptor set. With the largest Q^2^ and R^2^ values and the smallest RMSEV and RMSE values, models built with integrated descriptor sets were the best among all other models. 

### 2.3. Variable Importance Analysis

**Figure 2:**
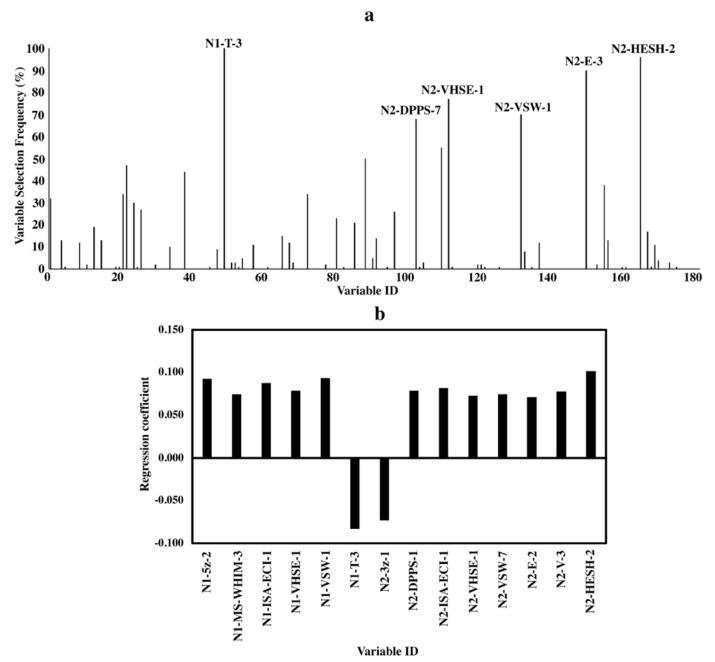
Variable importance of QSAR models for dipeptides. (**a**) Variable selecting frequencies of each variable from 100 BOSS runs; (**b**) standardized regression coefficients of each variable based on the model with the smallest RMSECV from 100 BOSS runs.

**Figure 3:**
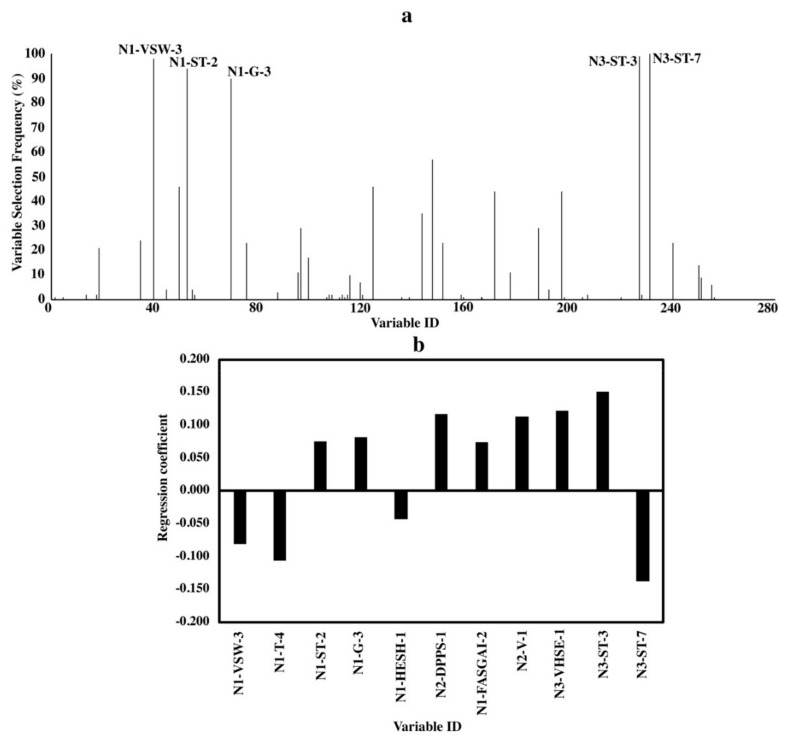
Variable importance of QSAR models for tripeptides. (**a**) Variable selecting frequencies of each variable from 100 BOSS runs; (**b**) standardized regression coefficients of each variable based on the model with the smallest RMSECV from 100 BOSS runs.

**Figure 4:**
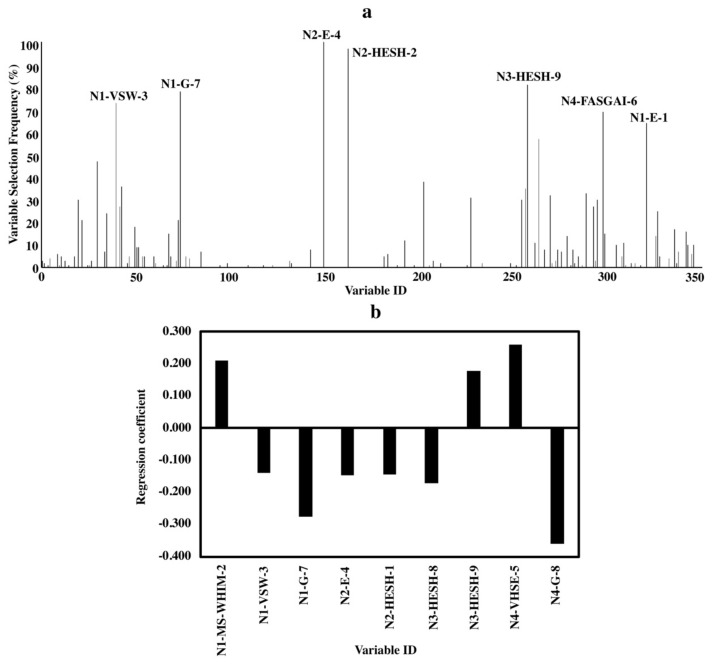
Variable importance of QSAR models for tetrapeptides. (**a**) Variable selecting frequencies of each variable from 100 BOSS runs; (**b**) standardized regression coefficients of each variable based on the model with the smallest RMSECV from 100 BOSS runs.

Variable importance analyses were done to elucidate the relationship between bitterness and the structure characteristics of amino acids in peptides. Due to the randomness of the algorithm of BOSS, variables selected in each run were slightly different [30]. Thus, the variable selecting frequency in 100 BOSS runs was combined with the standardized regression coefficient of the variables to analyze the variable importance. Results are shown in Figure 2, Figure 3 and Figure 4.

The standardized regression coefficient, which was calculated based on the standardized input (X) and output variables (Y), can quantify the relative importance of each input variables (X) because of the removal of the unit scale of variables during the standardizing treatment [30,31]. The variable is more important when the absolute value of the standardized regression coefficient is larger. For variable selecting frequency (Figure 2a, Figure 3a and Figure 4a), variable IDs of important variables with a variable selecting frequency of more than 60% were shown [30]. The equations of the models for di-, tri- and tetrapeptides with the smallest RMSEV obtained by 100 BOSS runs are shown in the Supplementary Table S4.

#### 2.3.1. Dipeptides

For dipeptides, an average of 12 independent variables were selected from the original 174 variables in each BOSS run.

Both variable selecting frequencies and standardized regression coefficients are shown in Figure 2a,b, respectively. Important variables selected by both methods consisted of different kinds of descriptor sets, which described various characteristics of both N- and C-terminal amino acids. The two variables with the highest selecting frequency were “N1-T-3” and “N2-HESH-2”, and they had the largest negative and positive standardized regression coefficients, respectively.

Although variable “N1-T-3” was selected through 100 BOSS runs, its specific structural characteristic of the amino acid indicated by “T-3” remains unknown [25]. The variable “N2-HESH-2” was selected 96 times and showed the largest positive impact on bitterness activity. This suggested the importance of high hydrophobicity of C-terminal amino acids in the dipeptides (Figure 2a,b). Similarly, other important variables like “N2-VHSE-1”, “N2-DPPS-1”, etc. also demonstrated the hydrophobicity of amino acids at the C-terminus. Besides the key role of a hydrophobic C-terminal amino acid in a dipeptide, both the size/bulkiness (in “N1-ISA-ECI-1” and “N1-5z-2”) and hydrophobicity (in “N1-VHSE-1”) of the N-terminal amino acids also showed positive correlations with bitterness in dipeptides.

#### 2.3.2. Tripeptides

Variable selecting frequencies and standardized regression coefficients for tripeptides are shown in Figure 3. On average, only 11 out of 261 independent variables were chosen from the BOSS runs.

Unlike the dominant role of the C-terminus to bitterness in the dipeptide, C- and N-terminuses in a tripeptide have nearly equal impact on bitterness with a weaker influence by the middle amino acids (Figure 3). Even though five variables with high selecting frequencies (>90%) were from “G”, “ST (Structural Topological)” and “VSW (Vector of Principle Components Scores for Weighted Holistic Invariant Molecular Index)” descriptor sets (Figure 3a), specific characteristics of the amino acids described by their parameters cannot be determined due to the fact that each parameter stands for a complex combination of different characteristics. At this stage, we only know that “G”, “ST” and “VSW” descriptors are related to physiochemical properties, topological structures and weighted holistic invariant molecular index, respectively [19,26,27]. From the standardized regression coefficient, we found some other important variables with clear information. For example, variable “N3-VHSE-1” reflects the hydrophobicity of C-terminal amino acids while “N2-DPPS-1” is related to the electronic properties of the middle-position amino acids (Figure 2b).

#### 2.3.3. Tetrapeptides

Figure 4 shows the results for tetrapeptides. Similar to tripeptides, only 12 out of 384 variables were screened out in each BOSS run on average.

Important variables for bitterness of tetrapeptides indicated the involvement of amino acids at all four positions (Figure 4a,b). Descriptors without clear indications are not discussed. For amino acids at N-terminus, both hydrophobic properties (N1-E-1) and size (N1-MS-WHIM-2) contributed to bitterness. The partial specific volumes (N2-E-4) and hydrophobic properties (N2-HESH-2) of amino acid at the second position were important for bitterness. Electronic properties were essential for both amino acids at the third position (N3-HESH-9) and C-terminus (N4-FASGAI-6 and N4-VHSE-5).

## 3. Discussion

QSAR models have been widely used in the study of peptide bioactivities [32,33,34]. Despite some models have been built before to predict bitterness in dipeptides, studies focusing on both tri- and tetrapeptides are very limited [5,15,20]. By using an integration of 14 amino acid descriptor sets combined with BOSS variable selection methodology, reliable bitter taste predicting models for di-, tri- and tetrapeptides are developed and reported here (Table 1 and Figure 1). To the best of our knowledge, this is the first report of building the QSAR models for bitterness of peptides based on integrated descriptor sets. Similar work was reported for dipeptides on the ACE-inhibition effect [30].

Even before variable selection, models for di-, tri- and tetrapeptides using integrated descriptor sets were better than most of the models using single set of amino acid descriptor (Table 2, Table 3 and Table 4), which are probably benefit from the more comprehensive descriptive information provided by the integrated descriptor sets and their mutual influences.

In this study, for dipeptides, models built with single set of amino acid descriptor showed good performance, with the best one belongs to the “VHSE” descriptor set. Other descriptor sets, namely, “DPPS”, “T-scale” and “G-scale”, were used for the modelling, which have never been reported before to predict bitterness in dipeptides. To compare models in this study with the previous ones, their R^2^, Q^2^ and RMSE were evaluated. Nevertheless, only slight differences were found in some of them, which could be explained by the differences in the number of principle components (A) used in the PLS regression. For example, in the “FASGAI” descriptor set, nine principle components were used in this study while three were reported in previous study [24]. Moreover, some studies applied other methods such as MLR [15] and SVM [35] to build their QSAR models, which may also contribute to different results. 

For tri- and tetrapeptides, only one work which used “3z-scales” combined with PLS regression is comparable to ours [5]. No models were built and reported with the remaining 13 single descriptor sets for both tri- and tetrapeptides. Even with the same datasets, descriptor sets, and number of principle components used, our results on tripeptides are quite different from theirs. We did not compare their RMSE values here because the published studies did not provide such values for comparison. The R^2^ and Q^2^ values obtained by us and the study by others were 0.503 and 0.385; 0.71 and 0.75, respectively. Similar situation is found for tetrapeptides. We used the principle component number of two while the reported study used four. We obtained R^2^ and Q^2^ of 0.822 and 0.490, respectively, for this study while from the literature; it showed 0.90 and 0.71, respectively, (Table 4). We speculated that these differences in results could be due to the different algorithms of the software used in the PLS regression and different methods used for cross-validation. In short, our Q^2^ results demonstrated much better predictability when models were built with a combination of 14 descriptor sets instead of one descriptor set (Table 3 and Table 4).

Variable selection is commonly used to eliminate the redundant descriptors in the development of QSAR models with larger number of variables to start with. The effectiveness of BOSS variable selection method has been proven before, and results also indicated its superiority to other methods like GA-PLS, CARS, and MCUVE [36]. After variable selection, the qualities of all three models for di-, tri- and tetrapeptides built with integrated descriptors were improved, especially for the predictive ability (Q^2^), suggesting the large contribution of BOSS variable selection process to the predictability of the models. Similar results were also obtained by Deng et al. 2017 [13], with an increase in R^2^ from 0.711 to 0.734 ± 0.004, and Q^2^ from 0.621 to 0.715 ± 0.002 for the model to predict the ACE-inhibition effect of dipeptides.

To elucidate the structure-bitterness relationship of di-, tri- and tetrapeptides based on our models, important variables obtained by the variable selecting frequency and the standardized regression coefficient were found to be nearly the same, indicating the reliability of the results (Figure 2, Figure 3 and Figure 4). Favorable results from both variable selecting frequency and standardized regression coefficient for all three models were not relied only on one descriptor set, but integrated descriptor sets.

Besides, the amino acids at different terminal locations in a peptide affected the behavior of the peptide differently (Figure 2, Figure 3 and Figure 4). This agreed with previous report which proposed that the N- and C-terminal locations of an amino acid residue in a sequence would determine the peptide bioactivity [37]. For example, the presence of high molecular weight C-terminal amino acids like Arg, Tyr and Lys favors the ACE-inhibition effects of dipeptides while for the N-terminus, amino acids with lower molecular weight and hydrophobic side chains like Leu are more preferred [6].

For the structure-bitterness relationship in dipeptides, the reported bitterness key role of the hydrophobic C-terminal amino acid remained unchanged even with the current 14 descriptor sets analyzed (Figure 2) [5], which further confirms its importance. For example, with a stronger hydrophobicity of C-terminal amino acid “F” [△f value (measurement of hydrophobicity) of 2650] than C-terminal amino acid “V” (△f value of 1690), dipeptide “AF” (19 mM) showed a lower bitter taste threshold than dipeptide “AV” (69 mM) [5,38]. Besides, bitterness was also contributed by the strong hydrophobic, polar/charged and large-size amino acids present at the N-terminus. For example, amino acids such as L, I and V are hydrophobic and have bulky side chains. Such relationship is consistent with other reported works [15,22].

For tripeptides, only bulkiness of amino acids at the N-terminus and hydrophobic amino acids at the C-terminus have been reported to correlate with bitterness before [5,15]. Despite the reported contribution of the hydrophobicity of the C-terminal amino acids (e.g., P, F, G, L, I) [5,15], we newly observed the contribution of the electronic properties of the middle amino acid, which was related to the first parameter of descriptor set “DPPS”, i.e., DPPS-1 (Figure 2 and Table S4). Larger DPPS-1 values of the amino acids in the middle position contribute to smaller bitterness thresholds of tripeptides. With a “DPPS-1” value of −2.86 and 2.34 for amino acids “G“ and “V”, respectively, the resulting bitterness threshold of tripeptide “GGV” (33 mM) was found to be larger than “GVV” (5 mM). Moreover, this study firstly demonstrated the importance of the “ST-scale”, “VSW” and “G-scale” descriptor sets to predict bitterness in tripeptides. Although the specific properties cannot be clearly elucidated due to the complex characteristics described by each single parameter in a descriptor set, they could still provide insights for future studies to further manifest the precise relationships.

For the tetrapeptides, only one study has described the structure-bitterness relationship before [5]. By comparing our results with theirs, we found that some important characteristics like the electronic properties of the C-terminal amino acids (N4-FASGAI-6 and N4-VHSE-5); hydrophobicity and size of the N-terminal amino acids (N1-E-1 and N1-MS-WHI-2) were the same (Figure 4). These indicate the importance of the presence of amino acids like F and P with hydrophobicity and bulkiness at the N-terminus. However, there are still some differences between our results and theirs, e.g., unlike their emphasis on the role of bulky hydrophobic C-terminal amino acids on bitterness, we found them less important in our study [5]. This could be due to the mutual influences among the different descriptor sets. Also, we found some important characteristics of amino acids that contribute to bitterness but have never been reported before, e.g., the partial specific volume (N2-E-4) which describes the 3D-structure of amino acids; the hydrophobic properties of the amino acid located at the second position (N2-HESH-2) and the electronic properties (N3-HESH-9) of amino acids at the third position (Figure 4 and Table S4). Amino acids with lower E-4 values at the second position of the tetrapeptides favors stronger bitterness in tetrapeptides, such as amino acids “R” (–0.258), “P” (–0.215) and “F” (–0.215) which have low E-4 values at the second position of the bitter tetrapeptides (Table S3). In addition, similar to tripeptides, involvement of the characteristics, described by “VSW” and “G-scale” descriptor sets (Figure 2, Figure 3 and Figure 4), provides useful information for future studies. In short, these findings not only confirm previous results but also provide additional properties which contributed bitterness in tetrapeptides.

In conclusion, three models built with integrated descriptors and BOSS variable selection method are highly reliable to predict bitterness of di-, tri- and tetrapeptides. The important structural characteristics of amino acids generated from comprehensive descriptive information for bitterness of di-, tri- and tetrapeptides were elucidated. These findings not only enhance our understanding of the bitterness-structure relationship of peptides but also provide more insights for future works. 

## 4. Materials and Methods

### 4.1. Preparation of Data Set

A total of 48, 52 and 23 dipeptides, tripeptides and tetrapeptides with bitterness activities were used. The bitter thresholds of these peptides were collected from different literatures [39,40,41,42,43,44,45,46,47,48,49,50] and summarized by Kim et al. [5]. The number of tetrapeptides used was small with only 23 because of the limitation in the available reported data. The details of the peptide sequences, the bitterness activities and the references were described in the Supplementary Tables S1–S3. The bitterness activity of a peptide is expressed as in Equation (1):

Bitterness activity = log (1/T),
(1)

where T is the bitter-tasting threshold concentration (M).

### 4.2. Independent Variables Used for Development of QSAR Model

In this study, the independent variables of the QSAR models were parameters of the amino acid-based descriptors. Fourteen descriptor sets describing the physio-chemical characteristics of amino acids were used, which included 3z-scale, 5z-scale, DPPS (Divided Physiochemical Property Scores), MS-WHIM-extended (Weighted Holistic Invariant Molecular approach applied on Molecular Surface), ISA-ECI (Isotropic Surface Area and Electronic Charge Index), VHSE (Principle Components Score Vectors of Hydrophobic, Steric, and Electronic Properties), FASGAI (Factor Analysis Scale of Generalized Amino Acid Information), VSW (Vector of Principle Components Scores for Weighted Holistic Invariant Molecular Index), T (Topological)-scale, ST (Structural Topological)-scale, E-scale, V, G-scale and HESH (Hydrophobic, Electronic, Steric, and Hydrogen). The number of parameters in each descriptor set was 3, 5, 10, 3, 2, 8, 6, 9, 5, 8, 5, 3, 8 and 12, respectively [11,12,13,14,18,19,21,22,23,24,25,26,27,28]. Thus, the total number of parameters of the 14 descriptor sets were 87.

The total number of independent variables were calculated by n x number of parameters, where ‘n’ is the number of amino acids in a peptide. For example, since the descriptor set “3z-scale” contained three parameters, when using the “3z-scale” to build the model for dipeptides, the total number of variables was six (i.e., 2 × 3). When using integrated descriptors to build a model for dipeptides, the total number of variables was 174 (i.e., 2 × 87) since the total number of parameters of the 14 descriptor sets was 87. Similarly, the total numbers of variables for tri- and tetrapeptides are 261 (i.e., 3 × 87) and 348 (i.e., 4 × 87), respectively, when using integrated descriptors.

Each independent variable was named in the following format: amino acid position-descriptor name-parameter number. For the amino acid position of dipeptides, N- and C-terminal ones are indicated by N1 and N2, respectively. For tripeptides, amino acid at the N-terminus, in the middle position, or at the C-terminus is indicated by N1, N2 and N3, respectively. Similarly, for tetrapeptides, the position is indicated by N1, N2, N3 and N4. For example, the first parameter of the descriptor set “3z-scale” for amino acid at N-terminus of a dipeptide is expressed as N1-3z-1.

### 4.3. Independent Variable Selection

A BOSS approach developed recently was used for variable selection in this study [36]. It is a combination of bootstrap sampling (BSS) [51], weighted bootstrap sampling (WBS) [52], model population analysis (MPA) [53] and PLS regression. The principles of these processes have been explained in detail by Deng et al. (2016) and will not be discussed here.

Briefly, the operation of BOSS approach contains four steps. Firstly, the bootstrapping sampling was applied to sample space to generate 1000 subsets and 1000 sub-models. Secondly, the prediction error indicated by the RMSECV of each model was calculated, and the best (10%) models with the lowest RMSECV were extracted. Thirdly, the regression coefficients of the independent variables in each extracted model were calculated and summed to obtain weights for each independent variable. Finally, a weighted bootstrapping sampling (WBS) was applied according to the new weights of variables to generate new subsets. Steps 2–4 were repeated until the number of independent variables in the new subsets equal to 1, and the subset with the lowest RMSECV during the iteration was chosen as the optimal variable set.

### 4.4. QSAR Model Building

The PLS regression method was used to build the QSAR model between the independent variables (X) described in Section 4.2 and the bitterness activities (log (1/T)) of di-, tri- and tetrapeptides. All data were autoscaled to unit variance before modeling.

The number of significant PLS components were chosen automatically by rules based on Q^2^ (the coefficient of determination of cross-validation). The goodness of model fit was estimated by R^2^ (coefficient of determination) and RMSE (Equation (2)). The standardized regression coefficient, which was calculated based on the standardized input (X) and output variables (Y) to remove the influence of the unit scale of variables, was used to evaluate the importance of input variables (X). The variable is more important when the absolute value of the standardized regression coefficient is larger.

(2) $$\mathrm{RMSE}=\sqrt{\frac{\sum_{i=1}^{N}{\left(y_{i}-\hat{y_{i}}\right)}^{2}\;}{N}},$$

where $y_{i}$ and $\hat{y_{i}}$ are the experimental and predicted bitter taste activities (i.e., Log(1/T)) of peptides. N is the number of peptides sets, 48 for dipeptides, 52 for tripeptides and 23 for tetrapeptides.

### 4.5. Model Validation

The developed models were assessed by 5-fold cross-validation as previously described [36], resulting in values for the RMSECV (Equation (3)) and the Q^2^ (the coefficient of determination of cross-validation; Equation (4)) [36].

(3) $$\mathrm{RMSECV}=\sqrt{\frac{\sum_{i=1}^{N_{Cal}}{\left(y_{i}-\hat{y_{i}}\right)}^{2}\;}{N_{Cal}}}$$

(4) $$Q^{2}=1-\;\frac{\sum_{i=1}^{N_{Cal}}{\left(y_{i}-\hat{y_{i}}\right)}^{2}\;}{\sum_{i=1}^{N_{Cal}}{\left(y_{i}-\bar{y_{i}}\right)}^{2}\;},$$

where $y_{i}$, $\hat{y_{i}}$ and $\bar{y_{i}}$ are the experimental, predicted and average predicted bitter taste activities (i.e., log(1/T)) of the peptides, respectively. $N_{Cal}$ is the number of calibration samples.

### 4.6. Statistical Analysis

All statistical analyses were performed by using MATLAB software (R2019a, The MathWorks, Inc., Natick, Massachusetts, USA).

## 5. Conclusions

With the integration of 14 descriptor sets, reliable QSAR models for predicting the bitterness of di- and tri- and tetrapeptides were built. They have the best fitting and predictability when compared with previous ones.

Using the variable importance analyzes based on both the variable selecting frequency and standardized regression coefficient, the key determinants for bitterness among different groups of peptides were elucidated. For dipeptides, the hydrophobic C-terminal amino acid played a dominant role followed by the contribution of a bulky hydrophobic amino acid at the N-terminus. For tripeptides, the hydrophobicity of C-terminal amino acids and electronic properties of amino acids at the second position were important. For tetrapeptides, bulky hydrophobic amino acids at the N-terminus; hydrophobicity and partial specific volumes of amino acids at the second position; and the electronic properties of amino acids at the remained two positions contributed to bitterness. 

In short, this study not only constructs reliable models for predicting the bitterness of di- tri- and tetrapeptides but also enhances better understanding of the structure-bitterness relationship of the peptides in each group and gives insights for their future studies.

## Figures and Tables

**Table 1:** Statistical parameters of quantitative structure–activity relationship (QSAR) models for di-, tri- and tetrapeptides using integrated descriptor sets.

BOSS ^a^	Variable Number	Name of Group	Statistical Parameters ^b^				
			A	R^2^	Q^2^	RMSECV	RMSE
No	174	Dipeptides	4.000	0.948	0.874	0.222	0.142
Yes	174	Dipeptides	2.000 ± 0.604	0.950 ± 0.002	0.941 ± 0.001	0.152 ± 0.001	0.139 ± 0.002
No	261	Tripeptides	3.000	0.760	0.521	0.407	0.289
Yes	261	Tripeptides	2.000 ± 0.450	0.770 ± 0.006	0.742 ± 0.004	0.299 ± 0.002	0.282 ± 0.004
No	361	Tetrapeptides	6.000	0.965	0.682	0.429	0.143
Yes	361	Tetrapeptides	6.000 ± 1.222	0.972 ± 0.002	0.956 ± 0.002	0.160 ± 0.004	0.127 ± 0.004

^a^ ‘Yes/No’ indicates the model was built with/without BOSS (bootstrapping soft shrinkage) variable selection process, respectively; ^b^ A: the number of principle components in PLS regression; R^2^: the coefficient of determination; Q^2^: the cross-validated R^2^; RMSECV: the root mean square error cross validation; RMSE: the root mean square error.

**Table 2:** Statistical parameters of QSAR models for dipeptides using a single set of amino acid descriptor and comparison with models built by integrated descriptor sets.

Descriptor	Variable Number	Statistical Parameters ^a^				
		A	R^2^	Q^2^	RMSECV	RMSE
3z-scale [11]	6	3	0.838	0.792	0.284	0.251
5z-scale [21]	10	5	0.916	0.869	0.225	0.180
DPPS [13]	20	5	0.934	0.849	0.242	0.160
MS-WHIM [22]	6	4	0.757	0.686	0.349	0.307
ISA-ECI [12]	4	2	0.845	0.808	0.273	0.245
VHSE [23]	16	7	0.943	0.894	0.202	0.149
FASGAI [24]	12	9	0.921	0.814	0.269	0.175
VSW [19]	18	4	0.911	0.773	0.297	0.185
T-scale [25]	10	6	0.900	0.830	0.257	0.197
ST-scale [26]	16	10	0.913	0.655	0.366	0.184
E-scale [14]	10	9	0.940	0.865	0.229	0.152
V [18]	6	5	0.904	0.863	0.231	0.193
G-scale [27]	16	9	0.937	0.855	0.238	0.157
HESH [28]	24	4	0.942	0.881	0.215	0.150
ID ^b^	174	4	0.948	0.874	0.222	0.142
ID + BOSS1 ^c^	174	2.000 ± 0.604	0.950 ± 0.002	0.941 ± 0.001	0.152 ± 0.001	0.139 ± 0.002
ID+BOSS2 ^d^	174	2	0.952	0.943	0.148	0.137

^a^ A: the number of principle components in PLS regression; R^2^: the coefficient of determination; Q^2^: the cross-validated R^2^; RMSECV: the root mean squares error cross validation; RMSE: the root mean squares error. ^b^ ID: integrated descriptor sets, which means a combination of all the 14 kinds of descriptor sets. ^c^ ID + BOSS1: integrated descriptor sets with BOSS (bootstrapping soft shrinkage) variable selection process, average statistical parameters of 100 runs. ^d^ ID + BOSS2: integrated descriptor sets with BOSS (bootstrapping soft shrinkage) variable selection process; statistical parameters for the model with the lowest RMSECV.

**Table 3:** Statistical parameters of QSAR models for tripeptides using a single set of amino acid descriptor and comparison with models built by integrated descriptor sets.

Descriptor	Variable Number	Statistical Parameters ^a^				
		A	R^2^	Q^2^	RMSECV	RMSE
3z-scale [11]	9	1	0.503	0.385	0.462	0.415
5z-scale [21]	15	2	0.669	0.526	0.405	0.339
DPPS [13]	30	5	0.722	0.444	0.439	0.310
MS-WHIM [22]	9	1	0.592	0.445	0.439	0.376
ISA-ECI [12]	6	1	0.525	0.357	0.472	0.406
VHSE [23]	24	3	0.689	0.439	0.441	0.329
FASGAI [24]	18	5	0.770	0.572	0.385	0.282
VSW [19]	27	5	0.789	0.504	0.415	0.270
T-scale [25]	15	1	0.629	0.375	0.465	0.359
ST-scale [26]	24	1	0.638	0.548	0.396	0.354
E-scale [14]	15	2	0.678	0.532	0.403	0.334
V [18]	9	2	0.560	0.432	0.444	0.390
G-scale [27]	24	6	0.745	0.533	0.402	0.298
HESH [28]	36	1	0.669	0.520	0.408	0.339
ID ^b^	261	3	0.760	0.521	0.407	0.289
ID + BOSS1 ^c^	261	2.000 ± 0.450	0.770 ± 0.006	0.742 ± 0.004	0.299 ± 0.002	0.282 ± 0.004
ID + BOSS2 ^d^	261	1	0.773	0.751	0.294	0.280

^a^ A: the number of principle components in PLS regression; R^2^: the coefficient of determination; Q^2^: the cross-validated R^2^; RMSECV: the root mean squares error cross validation; RMSE: the root mean squares error. ^b^ ID: integrated descriptor sets, which means a combination of all the 14 kinds of descriptor sets. ^c^ ID+BOSS1: integrated descriptor sets with BOSS (bootstrapping soft shrinkage) variable selection process, average statistical parameters of 100 runs. ^d^ ID+BOSS2: integrated descriptor sets with BOSS (bootstrapping soft shrinkage) variable selection process; statistical parameters for the model with the lowest RMSECV.

**Table 4:** Statistical parameters of QSAR models for tetrapeptides using a single set of amino acid descriptor and comparison with models built by integrated descriptor sets.

Descriptor	Variable Number	Statistical Parameters ^a^				
		A	R^2^	Q^2^	RMSECV	RMSE
3z-scale [11]	12	2	0.822	0.490	0.544	0.322
5z-scale [21]	20	6	0.938	0.533	0.521	0.189
DPPS [13]	40	8	0.968	0.676	0.433	0.136
MS-WHIM [22]	12	3	0.813	0.349	0.615	0.330
ISA-ECI [29]	8	3	0.717	0.017	0.755	0.406
VHSE [23]	32	4	0.922	0.694	0.421	0.213
FASGAI [24]	24	3	0.907	0.714	0.408	0.233
VSW [19]	36	6	0.969	0.512	0.532	0.135
T-scale [25]	20	1	0.624	0.452	0.564	0.467
ST-scale [26]	32	1	0.642	0.155	0.700	0.456
E-scale [14]	20	5	0.948	0.557	0.507	0.173
V [18]	12	2	0.794	0.525	0.525	0.345
G-scale [27]	32	4	0.879	0.620	0.469	0.265
HESH [28]	48	4	0.934	0.703	0.415	0.195
ID ^b^	348	6	0.965	0.682	0.429	0.143
ID + BOSS1 ^c^	348	6.000 ± 1.222	0.972 ± 0.002	0.956 ± 0.002	0.160 ± 0.004	0.127 ± 0.004
ID + BOSS2 ^d^	348	6	0.973	0.956	0.160	0.123

^a^ A: the number of principle components in PLS regression; R^2^: the coefficient of determination; Q^2^: the cross-validated R^2^; RMSECV: the root mean squares error cross validation; RMSE: the root mean squares error. ^b^ ID: integrated descriptor sets, which means a combination of all the 14 kinds of descriptor sets. ^c^ ID+BOSS1: integrated descriptor sets with BOSS (bootstrapping soft shrinkage) variable selection process, average statistical parameters of 100 runs. ^d^ ID+BOSS2: integrated descriptor sets with BOSS (bootstrapping soft shrinkage) variable selection process; statistical parameters for the model with the lowest RMSECV.

## Supplementary Materials

The following are available online, Table S1: Datasets of dipeptides; Table S2: Datasets of tripeptides; Table S3: Datasets of tetrapeptides; Table S4: Equations of QSAR models for di-, tri- and tetrapeptides with the smallest RMSECV from 100 BOSS runs.
