# Supplementary material for: Quantitative Structure–Activity Relationship Study of Bitter Di-, Tri- and Tetrapeptides Using Integrated Descriptors
Source: Molecules. 2019 Aug 5;24(15):2846. doi: 10.3390/molecules24152846 (PMC6696392; doi:10.3390/molecules24152846)
Supplement: Supplementary file 1 [file molecules-24-02846-s001.pdf]

Table S1. Dataset of dipeptides

Number	Sequence	log(1/T)	References	Number	Sequence	log(1/T)	References
1	GV	1.13	[40]	25	FG	1.77	[40]
2	AV	1.16	[40]	26	GY	1.77	[40]
3	VA	1.16	[40]	27	GF	1.80	[40]
4	VG	1.19	[40]	28	PY	1.80	[40]
5	PA	1.32	[40]	29	GW	1.89	[40]
6	GP	1.35	[40]	30	VL	2.00	[40]
7	ID	1.37	[40]	31	IV	2.05	[40]
8	IE	1.37	[40]	32	PL	2.22	[40]
9	IN	1.49	[40]	33	II	2.26	[40]
10	IQ	1.49	[40]	34	IL	2.26	[40]
11	IS	1.49	[40]	35	PI	2.33	[40]
12	IT	1.49	[40]	36	LL	2.35	[40]
13	SL	1.49	[40]	37	IP	2.40	[40]
14	WE	1.56	[40]	38	YL	2.40	[40]
15	IK	1.65	[40]	39	LY	2.46	[40]
16	GL	1.68	[40]	40	FP	2.70	[40]
17	IA	1.68	[40]	41	LF	2.75	[40]
18	IG	1.68	[40]	42	PF	2.80	[40]
19	AL	1.70	[40]	43	FL	2.87	[40]
20	GI	1.70	[40]	44	IW	3.05	[40]
21	VV	1.71	[40]	45	FF	3.10	[40]
22	AF	1.72	[40]	46	FY	3.13	[40]
23	LA	1.72	[40]	47	LW	3.40	[40]
24	LG	1.72	[40]	48	WW	3.60	[40]

Table S2. Dataset of tripeptides

Number	Sequence	log(1/T)	References	Number	Sequence	Log(1/T)	References
1	LGG	1.00	[38]	26	FFG	2.65	[36]
2	GGV	1.48	[42]	27	PPP	2.70	[36]
3	PGR	1.60	[36]	28	RPF	2.83	[36]
4	GPG	1.70	[46]	29	EGG	2.83	[45]
5	GYG	1.70	[39]	30	FIV	2.83	[42]
6	RGP	1.90	[36]	31	GGF	2.83	[36]
7	GLG	2.00	[38]	32	GGY	2.83	[39]
8	GGL	2.00	[38]	33	GLL	2.83	[38]
9	GGP	2.04	[46]	34	PIP	2.85	[47]
10	PGP	2.04	[46]	35	VIF	2.89	[39]
11	PPG	2.04	[46]	36	LLL	2.92	[38]
12	LLG	2.30	[38]	37	FGF	2.92	[36]
13	LGL	2.30	[38]	38	YGY	3.10	[39]
14	FGG	2.34	[36]	39	DLL	3.10	[36]
15	FPP	2.34	[39]	40	GRP	3.10	[45]
16	GVV	2.34	[41]	41	RPG	3.10	[36]
17	PGG	2.34	[39]	42	YYG	3.20	[39]
18	VVV	2.34	[39]	43	GFF	3.23	[36]
19	RRR	2.40	[44]	44	PFP	3.40	[46]
20	VYP	2.52	[46]	45	KPF	3.40	[39]
21	KPK	2.52	[46]	46	GYY	3.40	[39]
22	GFG	2.52	[36]	47	FPF	3.40	[39]
23	FPK	2.52	[39]	48	ELL	3.40	44
24	YGG	2.63	[39]	49	YPF	3.52	[46]
25	PPF	2.63	[39]	50	YYY	3.70	[39]
26	PGI	2.63	[46]	52	FFF	3.70	[36]

Table S3. Dataset of tetrapeptides

Number	Sequence	References	log(1/T)	Number	Sequence	Log(1/T)	References
1	GGLG	[38]	1.60	13	LLLL	3.23	[38]
2	GLGG	[38]	1.70	14	RPFG	3.41	[37]
3	LGGG	[38]	1.90	15	FGFG	3.52	[41]
4	GGGL	[38]	2.34	16	VYPF	3.52	[39]
5	PFPP	[46]	2.34	17	PFIV	3.52	[43]
6	FFGG	[44]	2.52	18	GPFF	3.80	[37]
7	FFPP	[39]	2.52	19	RGFF	3.80	[37]
8	GPPF	[39]	2.52	20	RPGF	3.80	[37]
9	RRPP	[44]	2.70	21	FGGF	3.92	[41]
10	FFPE	[44]	2.76	22	FFPR	4.00	[37]
11	GGFF	[44]	2.85	23	RPFF	4.40	[39]
12	FFPG	[44]	2.90				

Table S4. Equations of QSAR models for di-, tri- and tetrapeptides of the smallest RMSECV obtained by 100 BOSS runs

Name	Equation
Dipeptides[a]	$y = 0.0450X1 + 0.3069X2 + 0.0016X3 + 0.1197X4 + 0.0141X5 - 0.2338X6 - 0.0249X7 + 0.0318X8 + 0.0014X9 + 0.0813X10 + 0.0595X11 + 0.2551X12 + 0.0622X13 + 0.0964X14 + 1.4388.$
Tripeptides[b]	$y = -0.0358X1 - 0.1085X2 + 0.1825X3 + 0.1914X4 - 0.0520X5 + 0.0545X6 + 0.0561X7 + 0.5071X8 + 0.1435X9 + 0.2747X10 - 0.3066X11 + 2.3371$
Tetrapeptides[c]	$y = 0.4470X1 - 0.0695X2 - 0.1596X3 - 1.2383X4 - 0.2170X5 - 0.87895X6 - + 0.6643X7 + 0.3932X8 - 4.3698X9 + +5.04325.0432$

[a] Dipeptides: X1: N1-5z-2 ; X2: N1-MS-WHIM-3; X3: N1-ISA-ECI-1; X4: N1-VHSE-1; X5: N1-VSW-1; X6: N1-T-3 ; X7: N2-3z-1; X8: N2-DPPS-1; X9: N2-ISA-ECI-1; X10: N2-VHSE-1; X11: N2-VSW-7; X12: N2-E-2; X13: N2-V-3; X14: N2-HESH-2

[b] Tripeptides: X1: N1-VSW-3; X2: N1-T-4; X3: N1-ST-2; X4: N1-G-3; X5: N1-HESH-1; X6: N2-DPPS-1; X7: N1-FASGAI-2; X8: N2-V-1; X9: N3-VHSE-1; X10: N3-ST-3; X11: N3-ST-7

[c] Tetrapeptides: X1: N1-MS-WHIM-2; X2: N1-VSW-3; X3: N1-G-7; X4: N2-E-4; X5: N3-HESH-8; X6: N3-HESH-9; X7: N4-VHSE-5; X8: N4-G-8
